# Maternal and Fetal Outcome of Pregnant Patients Having Preexisting Cardiovascular Disease

**DOI:** 10.7759/cureus.9563

**Published:** 2020-08-05

**Authors:** Aamir Javaid, Abdul Majid, Shahida Aslam, Liaquat Ali, Muhammad Khalid Razaq, Syed Naseem I Bukhari, Anam Shaikh, Amber Rizwan

**Affiliations:** 1 Cardiology, Shaikh Zayed Medical College, Rahimyar Khan, PAK; 2 Cardiology, Nishtar Hospital Medical University, Multan, PAK; 3 Cardiology, Sheikh Zayed Medical College, Rahimyar Khan, PAK; 4 Cardiology, Chaudhry Pervaiz Elahi Institute of Cardiology, Multan, PAK; 5 Internal Medicine, Dow University of Health Sciences, Karachi, PAK; 6 Family Medicine, Dr. Ruth Pfau Hospital, Karachi, PAK

**Keywords:** adverse pregnancy outcome, cardiovascular disease, fetal outcome, maternal outcome

## Abstract

Introduction

Cardiovascular disease is common in woman of all age, including child bearing age. In this study, we aim to compare maternal and fetal outcome in pregnant woman with and without preexisting cardiovascular disease.

Methods

This case control single center study was conducted by Obstetrics & Gynecology department and Cardiology department Shaikh Zayed Medical College/Hospital Rahimyar Khan from 1^st^ March 2020 to 30^th^ June 2020.

Results

Pregnant woman with preexisting cardiovascular disease had more preterm births and newborn with lower birth weight. Maternal and fetal deaths were numerical higher in pregnant women with preexisting cardiovascular disease but statistically non-significant compared to woman without preexisting cardiovascular disease.

Conclusion

It is important to identify underlying cardiovascular disease in pregnant woman. Proper counselling throughout pregnancy is needed and efforts should be made to minimize risk of maternal and fetal complications.

## Introduction

In Pakistan, which is a developing country, heart diseases are not uncommon [1]. This is also the case in females of child bearing age. Common heart diseases in females include rheumatic/valvular heart disease, cardiomyopathy, diabetes and hypertension with their complications, and ischemic heart disease. Such diseases remain undiagnosed because of either ignorance or lack of healthcare facilities, which prevent timely screening and treatment before conception. Another factor contributing to their lack of early detection is the deceptiveness of symptoms and cardiac signs during pregnancy [2]. For example, valvular rheumatic heart disease is often only diagnosed during pregnancy because hemodynamic changes occurring as a result of the pregnancy uncover its symptoms [3]. And since a vast majority of these females are not aware of any preexisting heart conditions, many obstetrical complications occur, even resulting in death in 10% of cases [4]. According to a recent study, 15% of maternal deaths were caused by maternal heart disease [5].

In this study, we aim to determine the prevalence, type and characteristics of the heart disease, and the maternal outcomes of pregnancies complicated by heart disease, presenting to the labor room of Shaikh Zayed Medical College/Hospital, Rahimyar Khan, Pakistan. This study also aims to counsel these females for subsequent pregnancies, as well as advise them to counsel other women, thus potentially reducing the burden of disease over our developing society.

## Materials and methods

This case control single center study was conducted by Obstetrics & Gynecology department and Cardiology department Shaikh Zayed Medical College/Hospital Rahimyar Khan from 1st March 2020 to 30th June 2020. A total of 50 pregnant patients presented to the labor room with the history of cardiovascular disease (primary hypertension, arrhythmias and valvular disease) during this time duration were included in the study. Fifty pregnant women without cardiovascular disease were included in the study as part of control group. Thirty-six were booked and 14 were unbooked. Patients who developed cardiovascular disease during pregnancy were excluded from this study. Patients' informed consent was taken and their history, gestational age, blood pressure, mode of delivery, gravida and fetal weight were recorded in self-structured questionnaire. Maternal and fetal outcome was recorded as live or death in the same questionnaire. Ethical review was taken from Shaikh Zyed Medical College Institutional Review Board (IRB).

Data analysis was performed using IBM SPSS version 20 (IBM Corp., Armonk, NY). Numerical values such as maternal age, gestational age, BMI, blood pressure and fetal weight were presented as mean and standard deviation. Categorical data such as maternal and fetal outcome and mode of delivery was presented as frequencies. Mean were compared using t-test and frequencies were compared using chi-square. P-value less than 0.05 indicates that the difference between study and control group is significant enough to discard the null hypothesis.

## Results

The maternal age and blood pressure in case and control were comparable, however, control group had significantly higher gravida, gestational age at the time of giving birth and fewer cesarean section. Newborn weight was also significantly higher in control group (Table 1).

**Table 1:** Characteristics of Case and Control Group

Characteristics	Case (n = 50)	Control (n = 50)	P-value
Maternal Age (in years)	22 ± 6	23 ± 6	0.406
Gravida	2.2 ± 0.6	2.6 ± 0.7	0.002
Gestational Age (in weeks)	35 ± 4.2	37 ± 4.6	0.02
Blood pressure (mm/hg)			
Systolic	131.9 ± 19.7	126.9 ± 17.4	0.27
Diastolic	85.09 ± 11.2	84.09 ± 10.9	0.63
Mode of Delivery			
Vaginal	34 (68%)	47 (94%)	0.0001
Cesarean	16 (32%)	3 (6%)	
Newborn Weight (in kg)	2.01 ± 0.6	2.43 ± 0.8	0.003

Maternal and fetal deaths were numerical higher in pregnant women with preexisting cardiovascular disease but statistically non-significant compared to women without preexisting cardiovascular disease (Table 2).

**Table 2:** Maternal and Fetal Outcome

Characteristics	Case (n = 50)	Control (n = 50)	P-value
Maternal Death	3 (6%)	1 (2%)	0.3
Fetal Death	6 (12%)	1 (2%)	0.05

## Discussion

Maternal heart disease complicates at least 1% of pregnancies and is one of the most important causes of maternal death. Health problems may arise in expecting mothers due to hemodynamic burden and the hypercoagulable state of pregnancy [6]. In this study, we observed that a higher number of maternal and fetal deaths were associated with underlying cardiovascular disease.

Globally, there is limited data related to neonatal risk in pregnant women with existing cardiovascular disease. Still, it is a crucial component of antenatal care and plays a significant role in decision making leading to delivery of baby [7]. The ZAHARA (Zwangerschap bij Aangeboren HARtAfwijkingen) study identified twin/multiple gestations, smoking during pregnancy, cyanotic HD (corrected and uncorrected), mechanical valve prosthesis, and other cardiac medication before pregnancy as risk factors for adverse neonatal events [8].

In this study, pregnant women with underlying cardiaovascular disease were also found to be associated with premature birth and low infant birth weight. Both of these factors are known to pose an increased risk for complications in early life through adulthood, including but not limited to, respiratory issues, asthma, developmental delays, intestinal problems, infections, hearing loss, and retinopathy [9].

The mode of delivery in these women was decided by attending physicians after discussion with patients and primarily depended on obstetric indications and the maternal hemodynamic condition. Vaginal delivery was preferred in women with adequate cardiac output. However, in this study, a significantly higher proportion of women delivered via C-section. According to the European guidelines, primary Cesarean section should be considered for the patients on oral anticoagulants (OAC) in preterm labor, in women with severe heart failure, aortic root diameter >45 mm, and patients with acute or chronic aortic dissection [10].

An essential aspect of the assessment of reproductive-age women with cardiac disease is pre-conception counselling. A comprehensive assessment needs to cover the risks of pregnancy to both mother and foetus. Risks to the mother include whether she can tolerate the expected haemodynamic changes that occur in pregnancy and the potential long-term effects on the heart during and after pregnancy. Other aspects to consider are the need for highly medicalised antenatal care and delivery, and possibly a premature delivery, that may be far from home.

Appropriate prenatal counselling is critical for women with preexisting heart disease who are considering becoming pregnant because of the severe effects on the baby. This type of counselling is complex and should be done by an extensive team of physicians, including primary care and specialists, to optimally offer guidance and support to the patient. It requires expert advice on the assessment of maternal and fetal/neonatal risk of pregnancy, timing of pregnancy, and timing of pregnancy relative to future surgery or intervention. It also entails the management during and after pregnancy, labor and delivery, discontinuation of medications that may be teratogenic or continuation of medications that may prevent symptoms or complications during pregnancy, and proper knowledge of contraceptive choices appropriate for a patient's condition before the decision to become pregnant is made.

## Conclusions

In our study, pregnant women with preexisting heart disease were associated with preterm birth and low gestational weight. It was also associated with numerically increased maternal and fetal deaths. Based on the results above, it is really important to perform screening of pregnant women with pre-existing disease and ensure proper antenatal and post natal care is provided to them.

In summary, since preexisting heart disease is a significant contributing factor to both maternal and foetal health outcome, steps should be taken to offer optimal support to ensure minimal risk, and future mothers should be adequately counselled about the burden of disease on pregnancy.
